# Feasibility of Using Digital Clinical Records to Identify Factors Associated with Nursing-Sensitive Outcomes During Hospitalization: A Pilot Study

**DOI:** 10.3390/nursrep16090338

**Published:** 2026-09-17

**Authors:** Ivana Finiguerra, Maria Michela Gianino, Roberta Vacchelli, Marco Demasi, Lusi Pappalardo, Chiara Albanese, Elisa Binello, Giulia Borghin, Serena Cappello, Nadia Cavagnini, Serena Durante, Alice Ferraris, Fabiana Gorgonzola, Barbara Munaro, Stefania Pellegrino, Clara Russo, Laura Tedino, Davide Minniti, Salvatore Di Gioia

**Affiliations:** 1 Research and Innovation Unit for Health Professions, San Luigi Gonzaga University Hospital, 10043 Orbassano, Italy; i.finiguerra@sanluigi.piemonte.it (I.F.); m.demasi@sanluigi.piemonte.it (M.D.); l.pappalardo@sanluigi.piemonte.it (L.P.); c.albanese@sanluigi.piemonte.it (C.A.); e.binello@sanluigi.piemonte.it (E.B.); g.borghin@sanluigi.piemonte.it (G.B.); s.cappello@sanluigi.piemonte.it (S.C.); n.cavagnini@sanluigi.piemonte.it (N.C.); s.durante@sanluigi.piemonte.it (S.D.); a.ferraris@sanluigi.piemonte.it (A.F.); f.gorgonzola@sanluigi.piemonte.it (F.G.); b.munaro@sanluigi.piemonte.it (B.M.); s.pellegrino@sanluigi.piemonte.it (S.P.); c.russo@sanluigi.piemonte.it (C.R.); l.tedino@sanluigi.piemonte.it (L.T.); 2 Department of Public Health and Pediatrics, University of Turin, 10126 Torino, Italy; 3 Strategic Hospital Directorate, San Luigi Gonzaga University Hospital, 10043 Orbassano, Italy; d.minniti@sanluigi.piemonte.it (D.M.); s.digioia@sanluigi.piemonte.it (S.D.G.)

**Keywords:** nursing-sensitive outcomes, electronic health records, nurse staffing, patient safety, feasibility study

## Abstract

**Background/Objectives:** Nursing-sensitive outcomes are key indicators of nursing care quality and safety. Digitalization of clinical documentation enables prospective surveillance using routine data, but real-world evidence on integrated digital, clinical record-based approaches remains limited. This pilot study assessed the feasibility of using routinely collected digital data, combined with ward-level indicators, to support nursing-sensitive outcome surveillance and to preliminarily identify factors associated with these outcomes during hospitalization. **Methods:** An observational pilot study was conducted at a 400-bed Italian university hospital over two months (November 2025–January 2026) across ten wards. Clinical variables, including the Blaylock Risk Assessment Screening Score (BRASS) and ward-level indicators, were extracted from the electronic medical record system. The exploratory composite endpoint of documented nursing-sensitive outcomes was modeled using multivariable logistic regression with cluster-robust standard errors and Cox proportional hazards regression. **Results:** A dataset comprising 1552 hospitalizations and 20,415 patient-days was assembled, with 100% completeness for socio-demographic data and 93% for BRASS. The cumulative event rate of nursing-sensitive outcomes was 7.35% (6.08 per 1000 patient-days at risk; 5.58 per 1000 total patient-days). Pressure injuries were the most frequent event (*n* = 67), followed by falls (*n* = 39) and physical restraint (*n* = 14). In the adjusted models, the BRASS (OR = 1.12 per point, 95% CI 1.08–1.15; HR = 1.10, 95% CI 1.07–1.13) and admission to the hospital from the emergency department (OR = 4.41, 95% CI 3.02–6.45; HR = 3.46, 95% CI 2.48–4.83) were independently associated with nursing-sensitive outcome occurrence. In a 48 h landmark analysis restricted to hospitalized patients still event-free on day 2, the BRASS association was attenuated (OR 1.07, 95% CI 1.04–1.10). **Conclusions:** An integrated, digital, clinical record-based approach proved feasible for prospective nursing-sensitive outcome surveillance and supports the design refinements planned for longitudinal analyses.

## 1. Introduction

Nursing-sensitive outcomes are defined as patient outcomes that are at least partly influenced by the structure, processes, and quality of nursing care provided during hospitalization [1,2]. Within Donabedian’s framework of structure–process–outcome [3], nursing-sensitive outcomes play a crucial role in conceptualizing quality and safety in acute care settings. These outcomes have been progressively incorporated into both national and international quality monitoring systems, such as the National Database of Nursing Quality Indicators (NDNQI) and the indicators developed by the RN4CAST consortium [4,5]. Outcomes demonstrating sensitivity to nursing interventions across diverse healthcare settings have been classified into clinical, functional, safety, and perceptual domains [6].

Among safety nursing-sensitive outcomes, in-hospital falls, pressure injuries, and the use of physical restraints are recognized as prototypical events whose occurrence is widely considered indicative of the quality of nursing care provided at the ward level [7]. These three events hold considerable epidemiological importance, with incidence estimates of in-hospital falls ranging from 1 to 9 per 1000 patient-days [8,9]. Pressure injuries exhibit a global pooled incidence of 5.4 per 1000 patient-days and a prevalence of 12.8%, with substantial variability according to patient case-mix and setting [10]. Physical restraint, although characterized by substantial variability across countries and care settings, has been reported in 6% to 20% of hospital admissions in acute care [11,12]. All three events have been consistently associated with increased morbidity, prolonged length of hospital stay, higher healthcare costs, readmissions, and, in the case of falls and pressure injuries in particular, increased in-hospital mortality [13,14]. Reducing preventable adverse events associated with nursing-sensitive outcomes has been explicitly emphasized in the World Health Organization’s Global Patient Safety Action Plan 2021–2030 and is extensively integrated into healthcare quality metrics [15].

The factors influencing nursing-sensitive outcomes have been examined at two complementary levels. At the patient level, well-established risk factors include advanced age, functional dependence, cognitive impairment, comorbidities, and clinical instability at admission. These factors have led to the development of a broad spectrum of risk-stratification tools [16]. Since the early 2000s, extensive empirical research at the organizational level has consistently evidenced significant associations between nurse staffing levels, skill mix, and patient outcomes. Foundational contributions from Aiken and colleagues within the scope of the RN4CAST program [5,17], the research conducted by Needleman et al. [18], and the meta-analytical synthesis by Kane et al. [19] have collectively established that lower patient-to-nurse ratios and an increased proportion of nurses with university-level education are correlated with decreased rates of nursing-sensitive outcomes and mortality. More recent contributions further refined this understanding by emphasizing dynamic factors such as shift-level workload variability, the incidence of missed and omitted nursing care, and the influence of care transitions on the occurrence of nursing-sensitive outcomes. In the Italian context, multiple studies, including national contributions to the RN4CAST network and specialized research projects focusing on nursing workforce characteristics and patient outcomes, have supported the relevance of these associations and offered context-specific knowledge of the organization of nursing care within Italian acute care hospitals [20].

The methodological infrastructure for monitoring nursing-sensitive outcomes has historically depended on ad hoc data collection methods, such as standardized paper case report forms, prospective audits, and dedicated event registries. Although these approaches enable rigorous control over data quality and research design, they are resource-intensive, impose significant documentation burdens on clinical personnel, and are limited by scalability and sustainability concerns [21]. The transition to digital clinical documentation in hospitals has facilitated the use of routinely collected clinical workflow data for the prospective surveillance of nursing-sensitive outcomes, offering advantages in scalability, timeliness, and sustainability. Digital record-based surveillance has been increasingly implemented, particularly for outcomes like pressure injuries and healthcare-associated infections [22,23]. However, the integration of patient-level structured data with ward-level organizational indicators in unified analytical frameworks remains underdeveloped. Persistent methodological challenges include variability in the completeness and accuracy of structured documentation, reliance on documentation-based proxies that may increase under-reporting, especially for sensitive events, and mismatches between the temporal resolution of organizational indicators and the granularity needed for hypothesis testing. Furthermore, the integration of patient-, shift-, and ward-level data requires mature data architectures, which are still evolving in many healthcare settings [24]. Despite increasing methodological interest, empirical evidence regarding the operational and analytical feasibility of integrated digital record-based surveillance systems for nursing-sensitive outcomes in real-world acute care contexts remains sparse.

The hospital-wide shift from paper to digital clinical documentation in a large tertiary university hospital in northwestern Italy enabled an assessment of whether digital infrastructure can facilitate ongoing surveillance of nursing-sensitive outcomes and analysis of their associated factors. Prior to expanding to longitudinal evaluations, it was necessary to determine if integrating patient-level clinical data with ward-level indicators is feasible, if the planned analytical approach is applicable to the implemented digital clinical records, and what preliminary insights into key factors associated with nursing-sensitive outcomes can be identified.

Accordingly, this pilot study aimed to evaluate the feasibility of using routinely collected digital clinical data, integrated with ward-level organizational indicators, to enable prospective surveillance of nursing-sensitive outcomes during hospitalization. Additionally, the study sought to preliminarily characterize both patient- and ward-level factors associated with these outcomes within a real-world acute care context.

## 2. Materials and Methods

### 2.1. Study Design and Setting

This pilot observational study was conducted at the San Luigi Gonzaga University Hospital of Orbassano, Italy. The hospital is a 400-bed university hospital with a first-level Emergency Department (ED) and serves as a referral center for a large catchment area in the south-western part of the Metropolitan City of Turin and the Piedmont Region. The hospital employs approximately 1300 healthcare professionals and hosts the University of Turin’s San Luigi medical campus.

The original protocol for a longitudinal study evaluating nursing-sensitive outcomes comprised 12 months of prospective data collection across 20 wards using paper case report forms. However, the hospital’s transition to an upgraded electronic clinical record system prompted a pilot-phase study to evaluate the feasibility of using routinely collected digital clinical data and ward-level organizational indicators for monitoring nursing-sensitive outcomes and the factors associated with them. This article presents findings from the first two months of data collection (12 November 2025 to 12 January 2026) in ten hospital units, comprising half of those intended for the future longitudinal phase. Feasibility was assessed both operationally, regarding the capacity for automated, complete extraction and linkage of patient- and ward-level data without an additional burden on clinical staff, and analytically, based on whether the planned multivariable and time-to-event analyses could be applied to the digital dataset. These exploratory findings are intended to inform refinements of the subsequent longitudinal study design. The manuscript was drafted in accordance with the Strengthening the Reporting of Observational Studies in Epidemiology (STROBE) statement.

The ten participating wards were purposively selected from the twenty designated for the longitudinal phase, according to two predefined criteria. First, wards had to have fully implemented the updated electronic health record system prior to the pilot phase, as this constituted a technical prerequisite for automated data extraction. Second, both the medical and surgical departments needed to be represented, ensuring that the data architecture could be evaluated across a heterogeneous mix of cases and contextual profiles, including variations in size and patient-to-nurse ratios. Collectively, these ten wards comprised 252 of the hospital’s 400 beds.

### 2.2. Participants

Eligible participants were adult patients aged 18 years or older admitted to San Luigi Gonzaga University Hospital. Given the longitudinal design of the original study protocol, eligible patients were asked to provide written informed consent at admission. Patients who did not provide consent were excluded; their hospital record numbers were communicated to the ICT department, which removed the corresponding records, linked to a unique identification code, from the database. For patients unable to provide valid consent, consent was sought from a legal representative. Patients were excluded if they had an anticipated length of stay of 24 h or less in the emergency department observation unit, were admitted on a same-day or short-stay basis for outpatient surgical procedures, or had brief postoperative stays of 24 h or less in the intensive care unit.

### 2.3. Data Source and Digital Data Capture

The original study protocol involved collecting data through standardized paper case report forms completed at both the ward and individual patient levels. However, during the study planning phase, and concurrently with the hospital-wide upgrading of the electronic medical record system, the updated electronic platform was adopted as the primary source for patient- and admission-level data collection.

The electronic medical record system currently used at the hospital was introduced in 2024. It supports the administrative management of patient admissions by integrating diagnostic, clinical, and nursing care information within a single digital platform. In addition to storing clinical documentation, including emergency department reports, diagnostic test results, medical assessments, and specialist consultations, the system allows healthcare professionals to schedule treatments, plan care activities over a 24 h period, and create specific electronic records for falls, pressure injuries, and physical restraints when these events occur.

At the ward level, organizational data were collected from the electronic attendance system routinely used by each department. This system records bed occupancy and staffing levels for each of the three daily shifts (morning, afternoon, and night), with occupied beds typically recorded at 2:00 p.m. Ward-level organizational data were further integrated, at study start, with information on staff experience within the specific department and skill mix, including educational level (non-university-trained vs. university-trained nurses).

### 2.4. Outcome Definition

As originally planned in the study protocol, three nursing-sensitive outcomes were selected for monitoring: pressure injuries, falls, and physical restraint. In the present study, the primary endpoint was a composite nursing-sensitive outcome, defined as the occurrence of at least one of these events during hospitalization. An event was considered to have occurred when a specific digital record for pressure injury, fall, or physical restraint was created and linked to the patient’s unique identification code assigned at admission within the electronic medical record system. Two key aspects of the ascertainment process require explicit clarification. First, the electronic record is initiated upon documentation of any pressure injury, regardless of its temporal origin. During the study period, the electronic platform did not include a structured “present-on-admission” field; therefore, it was not possible to distinguish between pressure injuries present on admission and those acquired during hospitalization. The outcome measure was thus defined as documented pressure injuries of indeterminate origin. Because the electronic record only retained the calendar day, not the time, a pressure injury documented on the admission day cannot be distinguished from one that developed shortly after admission. This ambiguity applies only to pressure injuries, which may precede admission; in contrast, falls and physical restraint episodes are time-stamped and cannot occur before hospitalization. Thus, the composite endpoint may not represent events acquired during hospitalization and should not be interpreted as a direct measure of nursing care quality. Second, the use of physical restraint may be considered a nursing intervention rather than an unintended adverse event. It is included in this analysis as a nursing-sensitive outcome in accordance with international quality frameworks, which consider restraint use a safety indicator, with minimization reflecting the standard of nursing care [7]. Restraint is therefore included as a safety indicator rather than on the assumption that it shares a determinant structure with the other two components.

The decision to combine falls, pressure injuries, and physical restraint into a single composite endpoint was pragmatic. Over two months, these events were too infrequent to support multivariable analysis, so pooling was necessary to reach enough events for the models. The composite is exploratory, with the only common element being detection through the same electronic record linked to admission. We do not assert a common determinant structure across the components. Accordingly, the component-specific analyses presented in the results demonstrate that the measured patient-level factors are not consistently associated with all components. The components differ in clinical meaning, since physical restraint is a deliberate intervention, while falls and injuries are unintended. They also vary in preventability, as falls and restraint can be ward-prevented, but pressure injuries may reflect prior care. Lastly, they differ in timing: falls and restraint happen during admission, whereas a pressure injury is a clinical state that may precede hospitalization [15].

The unit of analysis was the hospitalization rather than the individual event. For the binary endpoint, each hospitalization contributed a single observation, which was coded as positive if at least one nursing-sensitive outcome of any type was documented during the hospital stay. In the case of the time-to-event analysis, only the first occurrence was modeled, with follow-up terminated at that point to ensure that no hospitalization contributed more than one event. This methodological approach was implemented to prevent a small subset of hospitalizations with recurrent outcomes from disproportionately influencing the estimates.

For analytical purposes, the endpoint was operationalized in two ways. First, it was treated as a binary outcome, indicating whether at least one nursing-sensitive outcome occurred during hospitalization. Second, it was treated as a time-to-event outcome, defined as the interval between hospital admission and the first recorded nursing-sensitive outcome. For patients who experienced an event, time to event corresponded to the recorded day of occurrence. For patients without an event, follow-up time corresponded to the total observed length of hospital stay.

### 2.5. Study Variables

Both nursing workforce and patient-level clinical variables were collected to investigate their association with nursing-sensitive outcomes. Nursing workforce variables encompassed the average number of registered nurses per shift and the average number of nursing assistants per shift, recorded for each of the three daily shifts. Additional variables included the patient-to-nurse ratio, professional experience, and educational level. The patient-to-nurse ratio was calculated using only registered nurses in the denominator, excluding nursing assistants, in accordance with the RN4CAST convention [5,17]. Throughout this manuscript, the term “nurse” refers specifically to registered nurses. Patient-level variables included age, sex, source of admission (waiting list, ED, direct access), and the Blaylock Risk Assessment Screening Score (BRASS). The BRASS was originally developed to identify patients at risk of prolonged hospitalization or in need of a structured discharge plan [25]. The scale was validated in Italian by Dal Molin et al. in 2014 and has been adopted as a mandatory screening tool in the Piedmont Region [26]. It includes 10 domains and yields a total score ranging from 0 to 40, with values of 0 to 10 indicating low risk, 11 to 19 moderate risk, and higher than 20 high risk. In the electronic medical record system of San Luigi Gonzaga University Hospital, the BRASS is included in the initial nursing assessment and is expected to be completed within the first 24 h after admission.

### 2.6. Data Collection Procedures

Clinical data at the individual patient level, together with ward-level information on staffing and bed occupancy, were collected through the newly implemented digital clinical documentation system and the hospital organizational data sources linked to it. At admission, each patient was assigned an administrative profile automatically linked to a unique identification code, which remained unchanged throughout their hospitalization. During each shift, ward nurses could access the patient’s digital profile to document care activities and, when appropriate, open specific electronic records to report falls, the presence of one or more pressure injuries, and the use of physical restraints.

Ward-level organizational data were entered by the chief nurse of each department through a dedicated electronic application, including the number of occupied beds and the number of nurses and nursing assistants present during the morning, afternoon, and night shifts. Information at the individual nurse level was collected through an electronic survey sent to institutional email addresses, inviting staff to participate through a secure online platform (response rate: 171/193 (88.6%); completeness: 100%). The survey collected ward-specific information on professional experience, educational qualification and age. Responses were aggregated to the ward level as the proportion of respondents with more than five years of experience in that ward, the proportion holding a university degree, and the mean age of the respondents. The denominator for the response rate was the number of registered nurses assigned to the ten participating wards at the start of the study period.

The final dataset was extracted with the support of the hospital IT engineering department. Data were retrieved for each patient identification code and linked to the discharge ward, which was used as the reference unit to integrate ward-level organizational variables with patient-level clinical data. The resulting database contained one row per hospitalization and included both individual clinical information and ward-level indicators for the observation period, such as average number of nurses per shift, average number of nursing assistants per shift, patient-to-nurse ratio, proportion of nurses with at least 5 years of ward-specific experience, and proportion of university-trained nurses. The extraction did not retain a stable patient identifier, so it could not be determined whether the 1552 hospitalizations corresponded to 1552 distinct individuals.

Organizational variables were linked to each hospitalization through the discharge ward, which was the only ward identifier available in the extraction: the source system did not retain the sequence of ward segments with their respective admission and discharge times, so the ward in which a patient was located at any given point of their stay could not be reconstructed.

This integrated data architecture enabled a preliminary assessment of whether routinely collected digital clinical and organizational records could support the prospective surveillance and exploratory risk modeling of nursing-sensitive outcomes.

### 2.7. Feasibility Criteria

Feasibility criteria were established a priori across two principal domains. Operational feasibility required (i) automated deterministic linkage of patient-level and ward-level data through the unique admission identifier, without manual record matching or manual transformation and without supplementary data entry by clinical staff; (ii) at least 95% completeness for essential structured fields related to sociodemographic and clinical variables; and (iii) an overall BRASS completeness rate of at least 90%, specified a priori as a sample-wide and not ward-level threshold. Analytical feasibility depended on (iv) a dataset sufficient to support multivariate association models, specifically with a minimum of ten events per variable, and (v) the ability to estimate both logistic and risk models, with formal verification of their underlying assumptions. All five criteria were required to demonstrate feasibility.

### 2.8. Statistical Analysis

Descriptive statistics were used to assess the completeness of digital clinical documentation and to synthesize patient characteristics, ward-level organizational variables, and the documented event rate of nursing-sensitive outcomes. Continuous variables were summarized using means and standard deviations (SDs), while categorical variables were reported as frequencies and percentages. The occurrence of outcomes was initially expressed as the cumulative event rate, defined as the proportion of hospitalizations in which at least one nursing-sensitive outcome was documented. Additionally, documented event rate density was calculated based on patient-days of observation. The documented event rate was calculated using the time at risk up to the first event as the denominator, consistent with the time-to-event analysis, while the corresponding figure based on the total hospital stay is reported alongside for comparison.

Bivariate analyses were conducted to investigate associations between the composite outcome and potential associated variables. Categorical variables were compared using Pearson’s chi-square test or Fisher’s exact test, as appropriate. Continuous variables were compared between patients with and without the outcome through logistic regression models or equivalent group comparison methods. For ward-level variables shared across multiple patients, robust standard errors clustered by ward were employed to account for intra-ward correlation. Standard errors were clustered at the ward level because organizational variables are constant within wards, so that hospitalizations in the same ward share exposure values and preventing anticonservative inference. With only ten clusters, the cluster-robust variance estimator is unreliable, so the effective independent observations are 10, not 1441. Two small-cluster procedures were used: score-based wild cluster bootstrapping with a null imposed (Rademacher weights, with exhaustive enumeration of all 2^10^ = 1024 sign combinations, so that no resampling variability arises), suitable for non-linear models, and referring the cluster-robust t statistic to a t distribution with G−1 degrees of freedom. Because all 1024 sign combinations were enumerated, the attainable two-sided *p*-values are multiples of 1/1024; the smallest attainable value is therefore 0.00098. For ward-level exposures, the wild cluster bootstrap is regarded as the primary inferential approach; conventional cluster-robust *p*-values are reported alongside for completeness and should not be interpreted independently.

To estimate adjusted associations with the binary outcome, multivariable logistic regression models were fitted, with the results expressed as odds ratios (ORs) and 95% confidence intervals (CIs). Given that key organizational variables were measured at the ward level, cluster-robust standard errors by ward were used in the primary multivariable analyses. Multivariable models were fitted on complete cases; missing BRASS values were addressed in a sensitivity analysis using multiple imputation by chained equations with 20 imputations. Model performance was assessed as apparent discrimination (area under the ROC curve), with a 95% confidence interval obtained by bootstrap percentile resampling of the dataset with the fitted model held fixed. Optimism and the optimism-corrected AUC were estimated separately, using 500 bootstrap resamples in which the model was refitted in each resample and evaluated on the original data. Calibration was assessed by the calibration slope, also optimism-corrected, and by the Hosmer–Lemeshow test. Time-to-event analyses were performed using Cox proportional hazards regression to estimate adjusted hazard ratios (HRs) with 95% CIs for the first occurrence of a nursing-sensitive outcome, with censoring applied to patients without an event at the end of follow-up. Hospitalized patients with zero recorded time at risk were assigned half a day of exposure, consistent with the convention used in the time-to-event models, ensuring that events on the day of admission stayed in the analysis. Both multivariable models were estimated via complete-case analysis for variables included in each respective model. Because discharge precludes the occurrence of an in-hospital event, it was treated as a competing event rather than as non-informative censoring. Cause-specific hazard regression was retained as the primary analysis, as the etiological question concerning the rate of occurrence among patients still at risk. Absolute risk was estimated separately using the Aalen–Johansen estimator, and a Fine–Gray subdistribution hazard model was fitted as a complementary analysis. The proportional hazards assumption was evaluated using Schoenfeld residuals, and the linearity of continuous variables on the log-hazard/logit scales was examined graphically. The assumption was satisfied for all covariates except the BRASS (χ^2^ = 15.83, *p* = 0.0001), whose effect was stronger in the early days of stay and attenuated thereafter; its hazard ratio is therefore interpreted as an average effect over the follow-up period. Four sensitivity analyses were conducted. Two focused on the composite endpoint: the first excluded cases in which only a pressure injury was documented on the admission day, and the second excluded events involving physical restraint. The third was a 48 h landmark analysis, including only hospitalizations still ongoing and event-free at day 2 to ensure events occurred after the BRASS assessment. The fourth restricted the sample by excluding hospitalizations with a ward transfer.

Component-specific models were fitted according to a predefined criterion based on the number of events per variable: the full eight-parameter model when at least five events per variable were available; a reduced model containing age, admission source, and BRASS score when this threshold was met with only four parameters; and descriptive reporting alone when neither model reached it. No a priori power calculation for hypothesis testing was performed because the study was designed as a feasibility pilot. Its primary objectives were to assess data completeness, linkage feasibility, and analytical tractability rather than to estimate a prespecified effect size. Therefore, the sample size was determined by the study design, and all consecutive eligible admissions to the ten participating wards during the two-month study period were included. The events-per-variable criterion addresses model estimability only and does not establish the reliability of individual estimates, particularly for ward-level terms, whose effective sample size is the number of wards. Descriptive and regression analyses were conducted using IBM SPSS Statistics, version 29.0. Wild cluster bootstrapping, competing-risk analyses (Aalen–Johansen and Fine–Gray), multiple imputation and bootstrap internal validation were performed in R version 4.4.1 (packages sandwich, cmprsk, mice, survival and rms). All statistical tests were two-sided, and *p*-values < 0.05 were deemed statistically significant.

### 2.9. Ethical Considerations

The study protocol specified that the study was observational, based on pseudoanonymized data, and to be conducted in accordance with the Declaration of Helsinki and applicable privacy regulations. It was approved by the Ethics Committee of the Città della Salute e della Scienza of Turin, Italy (protocol code 438/2025).

## 3. Results

### 3.1. Data Completeness and Patient- and Ward-Level Characteristics

**Figure 1:**
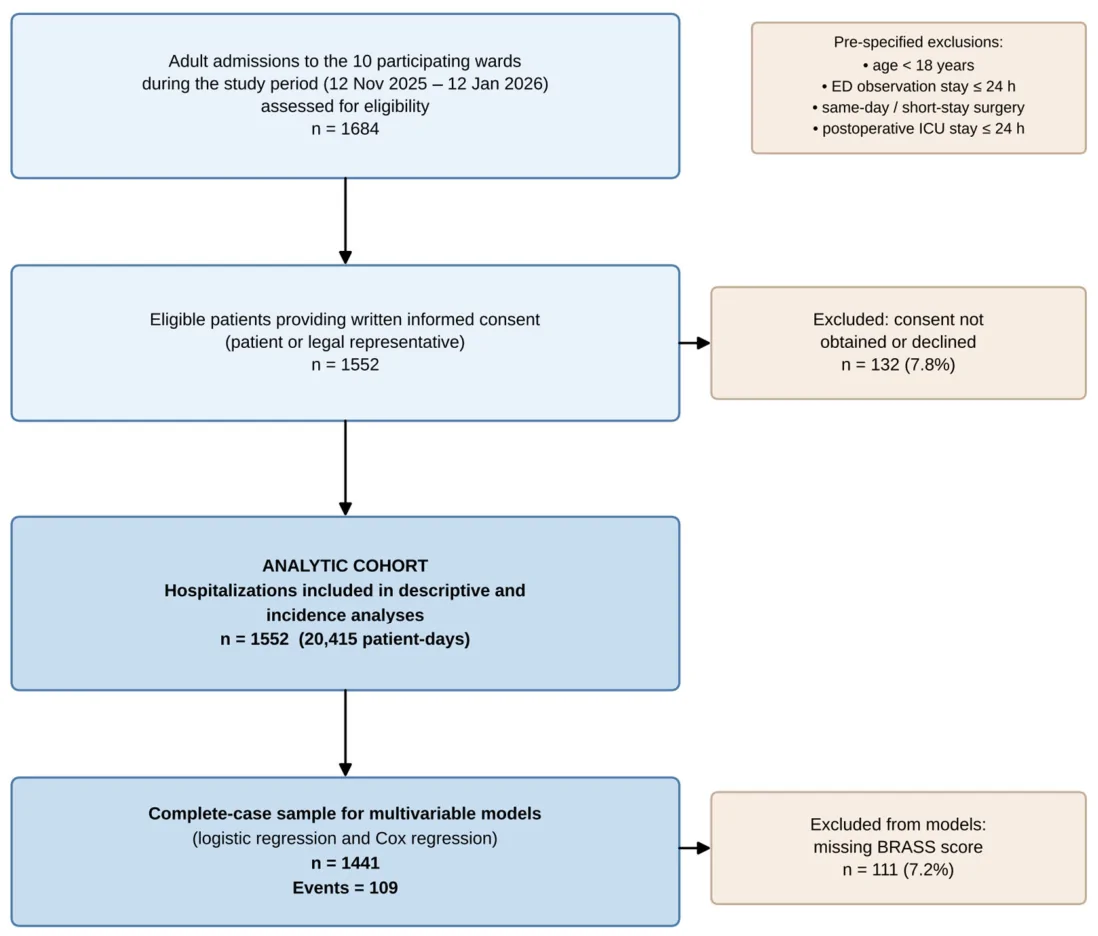
Participant flow diagram.

Of 1684 eligible patients admitted during the study period in the ten included wards, 132 (7.8%) did not provide consent and were removed from the database, yielding the analytic cohort of 1552 hospitalizations, for a total of 20,415 days of observation (Figure 1).

Age, sex, admission source, ward, length of stay and outcome status were complete for all 1552 hospitalizations. The BRASS was missing for 111 hospitalizations (7.2%), and completeness varied markedly by ward, from 79.3% to 100%. Since the BRASS is a mandatory regional assessment, this range identifies documentation practice rather than data availability as the principal determinant of completeness and thus a modifiable target for the surveillance system rather than an intrinsic limitation of the digital architecture (Table 1). Completeness varied markedly by ward, ranging from 79.3% to 100%. Included patients had a mean age of 71.4 years (SD ± 14.4); 62.1% were male (*n* = 964).

The included units had bed capacities ranging from 18 to 31, occupancy rates from 80% to 100%, and patient-to-nurse ratios from 6.86 to 14.07 (Table 2). Nurses had a mean age of 44 years; the proportion of nurses with more than five years of ward-specific experience was 57%, while the proportion of university-trained nurses was 64%.

Length of stay was markedly right-skewed (skewness 5.02), with a median of 7 days (IQR 3–16). It varied substantially across wards, with ward medians ranging from 3 to 50 days, and between areas, with a median of 12 days in the medical area and 5 days in the surgical area. Ward transfers occurred in 241 of the 1552 hospitalizations (15.5%), with up to eight transfers recorded for a single admission.

### 3.2. Study Population and Event Rate of Nursing-Sensitive Outcomes

Throughout the study period, a total of 114 hospitalizations involved at least one nursing-sensitive outcome, resulting in a cumulative documented event rate of 7.35%. In particular, six hospitalizations involved multiple nursing-sensitive events, including three cases of falls with physical restraint, two falls with pressure injuries, and one case involving both physical restraint and pressure injuries. The most frequently observed nursing-sensitive outcome was pressure injury (*n* = 67; 4.32%), followed by falls (*n* = 39; 2.51%), and physical restraint (*n* = 14; 0.9%). Importantly, 32 (48%) of the pressure injuries were documented on the day of admission, with a median recording day of 1. In contrast, the median recording day was 9 for falls and 3 for physical restraints.

### 3.3. Distribution of Nursing-Sensitive Outcomes

The documented event rate of nursing-sensitive outcomes varied significantly across hospital departments. Within the medical department, 81 of 834 hospitalizations (9.71%) had at least one event, whereas in the surgical department, 33 of 718 hospitalizations (4.60%) were affected. Statistical analysis confirmed the significance of this difference (χ^2^ = 14.10, *p* < 0.001). When expressed per unit of time at risk, the overall event rate density was 6.08 per 1000 patient-days (5.58 per 1000 total patient-days of hospital stay). In contrast to the cumulative event rate, rate density did not differ significantly between the medical and surgical departments (Table 3).

The cumulative documented event rate differed significantly across individual wards, ranging from 0.89% in Urology to 20.75% in Geriatrics, with elevated rates also observed in Internal Medicine (14.84%) and Orthopedic Surgery (10.78%) (χ^2^ = 75.56, *p* < 0.001). Unlike the department-level comparison, ward-level differences persisted after standardization per patient-days (*p* < 0.001).

Composite documented nursing-sensitive outcomes also varied according to several sociodemographic, clinical, and organizational characteristics. The occurrence of composite documented nursing-sensitive outcomes was higher among female than male patients (10.7% vs. 5.3%) and increased with older age and higher BRASS. In addition, wards with higher mean bed occupancy rates and higher patient-to-nurse ratios showed a greater occurrence of composite documented nursing-sensitive outcomes (Table 4). Composite documented nursing-sensitive outcomes occurred in 9.13% of transferred patients and 7.02% of those remaining in a single ward, with no significant difference (*p* = 0.248).

### 3.4. Bivariate Associations with Organizational and Clinical Variables

Among the organizational variables assessed at the ward level, patients who experienced composite documented nursing-sensitive outcomes were more frequently admitted to wards characterized by a higher mean bed occupancy rate (0.950 ± 0.061 vs. 0.910 ± 0.076). Logistic regression analysis, utilizing ward-clustered robust standard errors, demonstrated that each 0.10 increment in mean bed occupancy rate corresponded to significantly increased odds of outcome occurrence (OR = 2.28, 95% CI: 1.01–5.14, *p* = 0.048).

No statistically significant association was identified between the mean number of nurses per shift and the occurrence of composite documented nursing-sensitive outcomes (2.57 ± 0.28 vs. 2.61 ± 0.24; OR = 0.94 per 0.10 increase, *p* = 0.519). In contrast, patients who experienced outcomes were more frequently discharged by wards with a higher mean number of nursing assistants per shift (1.80 ± 0.34 vs. 1.58 ± 0.32; OR = 1.24 per 0.10 increase, 95% CI: 1.11–1.38, *p* < 0.001).

Regarding skill-mix indicators, the mean patient-to-nurse ratio was notably higher among patients who experienced composite documented nursing-sensitive outcomes (10.33 ± 2.55 vs. 9.23 ± 1.96). Furthermore, each additional patient per nurse was significantly associated with increased odds of outcome occurrence (OR = 1.24, 95% CI: 1.07–1.44, *p* = 0.004). In contrast, no statistically significant associations were identified for the proportion of nurses with at least five years of ward-specific experience or the proportion of university-trained nurses.

At the patient level, those who experienced composite documented nursing-sensitive outcomes were, on average, older than those without such outcomes (80.3 ± 12.2 years vs. 70.7 ± 14.4 years), and age showed a robust association in univariable analysis (OR = 1.07 per year, *p* < 0.001). Furthermore, event rates were significantly higher in women than men (10.7% vs. 5.3%; χ^2^ = 15.00, *p* < 0.001; crude OR = 2.15, 95% CI: 1.46–3.16). As a result, although the cohort was predominantly male (62.1%), women nonetheless accounted for 55.3% of all patients experiencing an outcome despite being the minority sex overall.

The source of patient admission emerged as one of the strongest crude correlates of composite documented nursing sensitive-outcome occurrence. Specifically, the observed event rates were 1.2% for individuals admitted from waiting list, 5.2% for those classified as direct access, and 11.9% for those admitted from the emergency department (χ^2^ = 58.26, *p* < 0.001). Relative to admissions from waiting list, the unadjusted odds of the nursing-sensitive outcome were substantially elevated for both direct access and Emergency Department admissions.

The BRASS showed a strong association with the studied endpoint. Individuals who experienced nursing-sensitive outcomes showed markedly higher BRASS values compared to those without such outcomes (16.18 ± 8.37 versus 7.21 ± 6.75). Univariable logistic regression analysis indicated that each one-point increment in the BRASS corresponded to a 14% increase in the odds of nursing-sensitive outcome occurrence (OR = 1.14, 95% CI: 1.11–1.17, *p* < 0.001).

### 3.5. Multivariable Logistic Regression

The presence of any composite documented nursing-sensitive outcome was included as the dependent variable in the multivariable logistic model, where structural and clinical variables were included as independent variables (Table 5). Structural variables included the department (medical/surgical), mean bed occupancy rate, and patient-to-nurse ratio. Clinical variables were age, sex, admission source, and BRASS. Due to BRASS missing data for 111 hospitalizations, the complete-case analysis comprised 1441 hospitalizations, among which 109 patients experienced a nursing-sensitive outcome.

In the adjusted model, two variables (admission source and BRASS) remained independently associated with composite documented nursing-sensitive outcome occurrence. Compared with waiting lists, the odds of nursing-sensitive outcome occurrence were higher in patients admitted for direct access (OR = 4.15, 95% CI: 1.66–10.39, *p* = 0.002) and to the emergency department (OR = 4.41, 95% CI: 3.02–6.45, *p* < 0.001). The BRASS continued to demonstrate an independent association, with each one-point increment conferring a 12% increase in the odds of event occurrence (OR = 1.12, 95% CI: 1.08–1.15, *p* < 0.001).

Following adjustment, the department association was not supported once the small number of clusters was accounted for, with the surgical department showing greater odds than the medical department (OR = 5.67, 95% CI 0.97–33.10, conventional *p* = 0.054; wild cluster bootstrap *p* = 0.135). Mean bed occupancy rate, patient-to-nurse ratio, age, and sex were not associated with nursing-sensitive outcomes in the adjusted model. Apparent discrimination was 0.828 (bootstrap 95% CI 0.788–0.863). Internal validation with 500 bootstrap resamples gave an optimism of 0.013 and an optimism-corrected AUC of 0.816 (95% CI 0.783–0.851). Calibration was adequate, with an optimism-corrected calibration slope of 0.952 and a non-significant Hosmer–Lemeshow test (χ^2^ = 4.07, df = 8, *p* = 0.851).

### 3.6. Time-to-Event Analyses

In the multivariable Cox proportional hazards model, the same set of covariates as utilized in the logistic regression analysis was included (Table 6). In the fully adjusted model, direct-access admission (HR = 2.59, 95% CI: 1.32–5.09, *p* = 0.006), emergency department admission (HR = 3.46, 95% CI: 2.48–4.83, *p* < 0.001), and BRASS (HR = 1.10 per one-point increment, 95% CI: 1.07–1.13, *p* < 0.001) showed independent associations with an elevated risk of experiencing the first nursing-sensitive outcome. Also in this case, the departmental association was not statistically supported once the small number of clusters was accounted for, with the surgical department showing a higher adjusted hazard compared to medical department (HR= 3.81, 95% CI: 0.83–17.56, *p* = 0.087; wild cluster bootstrap *p* = 0.225). Notably, mean bed occupancy rate, patient-to-nurse ratio, age, and sex were not significant in the Cox model. Treating discharge as a competing event, the cumulative event rate of a documented nursing-sensitive event was 5.41% at day 7, 6.18% at day 14, and 7.15% at day 30, converging on the crude proportion of 7.56%. The Fine–Gray model yielded subdistribution hazard ratios close to the cause-specific estimates (BRASS 1.11, Emergency Department admission 4.17).

### 3.7. Sensitivity and Component-Specific Analyses

Excluding hospitalizations with only admission-day pressure injuries, cases decreased from 114 to 82 (from 109 to 77 in the complete data). The BRASS’s OR decreased from 1.12 (95% CI 1.08–1.15) to 1.05 (95% CI 1.02–1.08), and discrimination declined from 0.828 to 0.779, while the association with admission from the Emergency Department was unchanged. In the 48 h landmark analysis with 1247 ongoing, event-free hospitalizations at day 2, the BRASS OR was 1.07 (95% CI 1.04–1.10), discrimination 0.814. Excluding transfers or physical restraints did not significantly change patient associations. The department term remained imprecise across all analyses, with confidence intervals spanning one to two orders of magnitude (Table 7). Because the time of BRASS completion was not retained, this restriction approximates rather than establishes temporal precedence.

Of the 114 hospitalizations with a documented event, 45 (39.5%) had the event on admission day, 54 (47.4%) within the first day, and 60 (52.6%) from day 2 onwards. For nursing-sensitive outcomes: 32 of 67 pressure injuries, 10 of 39 falls, and 4 of 14 physical restraints occurred on admission. Pressure injuries accounted for 67 of 114 hospitalizations with a nursing-sensitive outcome, representing the major contributor to the composite endpoint.

Compared with pressure injuries documented from day 1 (*n* = 35), those on admission day (*n* = 32) occurred in patients with a more compromised risk profile: median BRASSs of 23.5 versus 13.5 (*p* < 0.001) and 96.9% versus 82.9% admitted from the emergency department (*p* = 0.108). The median stay was shorter (12 versus 22 days, *p* = 0.031), and age was similar (83 versus 84 years, *p* = 0.768). These differences could indicate pressure injuries present at admission or rapid injury development in high-risk patients.

Excluding physical restraint from the composite (99 events) left both patient-level associations unchanged (BRASS OR 1.12, 95% CI 1.09–1.15; emergency department OR 3.52, 95% CI 2.28–5.43). In the same way, excluding the 241 transferred hospitalizations (1229 hospitalizations, 89 events) left them unchanged (BRASS OR 1.11, 95% CI 1.08–1.15; emergency department OR 3.59, 95% CI 2.27–5.68), whereas the department estimate became nominally significant but remained unsupported under small-cluster correction (OR 11.00, 95% CI 1.03–117.67; wild cluster bootstrap *p* = 0.148).

Component-specific analyses (Table 8) showed that the measured patient-level factors were not uniformly associated with the three components. The BRASS was strongly associated with pressure injuries (OR 1.16, 95% CI 1.12–1.21) but only marginally with falls (OR 1.05, 95% CI 1.00–1.10). Admission from the emergency department showed the reverse pattern (falls OR 5.87, 95% CI 3.19–10.81). Physical restraint, with 14 events, could not support a multivariable model and is reported descriptively. Six of the 114 hospitalizations with a documented event (5.3%) involved more than one component.

Hospitalizations without a BRASS assessment differed from those with one in age and sex but not in outcome frequency, admission source or length of stay (Supplementary Table S1). Multiple imputation across the full sample of 1552 hospitalizations reproduced the complete-case estimates (BRASS OR 1.12, 95% CI 1.09–1.15; emergency department OR 4.67, 95% CI 3.21–6.80).

## 4. Discussion

This pilot study aimed to evaluate the feasibility of using routinely collected digital clinical and organizational data for the identification and analysis of composite documented nursing-sensitive outcomes during hospitalization. Additionally, it sought to provide a preliminary characterization of the factors associated with them within a real-world acute care setting. Our findings showed the operational and analytical feasibility of an integrated digital architecture, while organizational effects could not be reliably estimated owing to the aggregation of staffing measures over the study period and the limited number of wards. The findings of this pilot study provide insights for the development of digital health monitoring systems, in line with current international policy.

The integrated extraction achieved 100% completeness for sociodemographic and clinical data and 93% for the BRASS. Completeness of the BRASS ranged from 79.3% to 100% across wards, indicating that documentation practice, rather than data availability, is the main constraint on completeness and a specific target for the longitudinal phase. No additional documentation tasks were imposed on ward nurses, although workforce variables required an electronic staff survey and the extraction required information-systems support. The hospital information systems department executed extraction queries and validated the output; linkage was deterministic and automated, requiring no manual matching. Extraction was performed on request, not automated, so the system cannot not yet provide real-time surveillance. This operational outcome is relevant to the WHO’s recommendations on safe nursing staffing [27], which call for information systems that link staffing data to patient outcomes without burdensome manual processes and which identify indicators such as the nurse-to-patient ratio, length of stay and rates of nursing-sensitive outcomes as a basis for benchmarking. The architecture demonstrated here generates those indicators, but it is an integrated extraction system requiring institutional information-systems support rather than the fully automated real-time surveillance envisaged in those recommendations, which remains the objective of the longitudinal phase. Two patient-level variables were independently associated with nursing-sensitive outcomes in both the logistic regression and Cox proportional hazards models: the BRASS and the source of admission. In contrast, ward-level organizational indicators, specifically the bed occupancy rate and patient-to-nurse ratio, which exhibited significant crude associations in unadjusted analyses, did not retain statistical significance after adjustment for case mix.

The BRASS was initially developed and validated as a tool to identify patients at risk of prolonged hospitalization or requiring a structured discharge plan [25,26]. Although its utility for stratifying risk of nursing-sensitive outcomes has not been thoroughly investigated, the associations identified in this study (adjusted OR = 1.12 and HR = 1.10 per one-point increase) warrant attention. A plausible explanation for this finding is the multidimensional nature of the scale, which includes parameters such as age, cognitive status, functional deficits, mobility limitations, comorbidities, and social support. These domains overlap with recognized risk factors for these events [28,29], although the association was confined to pressure injuries and was only marginal for falls. In this sense, the BRASS may function as a composite proxy of frailty rather than solely as a discharge-planning instrument. The components, however, overlapped in only 5.3% of affected hospitalizations and are not interchangeable. Thus, the BRASS is most accurately characterized as a variable associated with outcome occurrence, and potentially as a baseline indicator of risk, rather than as a validated prospective predictor; the present study design does not allow for confirmation of the latter. Its potential for this role should be assessed through longitudinal studies over extended observation periods. Beyond our findings, the three nursing-sensitive outcomes vary in their likely causal pathways and in the degree to which they can be attributed to nursing care during the observed stay. A fall or an episode of restraint occurs at a specific moment during admission, whereas a pressure injury represents a clinical state that may have preceded the admission. Only 6 of the 114 hospitalizations with an event (5.3%) involved more than one component, indicating that the composite predominantly aggregates distinct groups of hospitalizations rather than a single, homogeneous construct.

The association between the BRASS and the composite endpoint was attenuated in sensitivity analyses. This may explain why part of the main model may reflect a current state rather than prospective risk, as roughly half of the events did not have a preceding BRASS. Excluded events were concentrated in the upper BRASS tail; removing them narrowed exposure contrast and reduced the estimated effect for reasons of composition. Still, the association remained significant, and the departure from proportional hazards for BRASS reflects its static measure at admission as a proxy for a changing state. BRASS captures a real risk dimension, but its early stratification tool cannot be confirmed from a study where assessment and event are not reliably ordered over time. Retaining the assessment timestamp in the longitudinal phase constitutes a planned methodological enhancement. However, it does not address the temporal ambiguity inherent in the current analysis. Admission from the ED was associated with a more than fourfold increase in the likelihood of nursing-sensitive outcomes compared with elective admissions from the waiting list (adjusted OR = 4.41; HR = 3.46). This variable showed the strongest individual association across both multivariable models and was the dominant factor for falls in component-specific analysis. These findings align with the broader literature documenting higher rates of nursing-sensitive outcomes associated with unplanned admissions, which typically involve older, more clinically unstable patients who are often admitted with incomplete information about comorbidities and functional status [30]. In particular, time spent in the emergency department has been linked to delays in initiating preventive nursing interventions and to an increased incidence of in-hospital complications [31]. The intermediate risk profile of the direct-access group (OR = 4.15; HR = 2.59) is plausibly explained by clinical deterioration during outpatient follow-up: these patients approach the acuity of those admitted through the ED yet bypass ED boarding and its associated care delays, which may account for their somewhat lower risk.

The crude, ward-level organizational indicators observed in this pilot study are consistent with the extensive body of organizational research [17,18,19] that links staffing levels to clinical outcomes. Notably, there is a documented association between higher patient-to-nurse ratios and nursing-sensitive outcomes, including increased patient mortality. In our analysis, these associations were statistically significant in univariable models but were substantially attenuated after adjusting for case-mix. This pattern underscores a key message of the WHO recommendations: staffing adequacy should not be reduced solely to headcount or a fixed numerical ratio alone. A ward that appears fully staffed in numerical terms may still carry a hidden skills deficit, and variations in patient clinical complexity, competencies, and case-mix must be explicitly incorporated into staffing decisions.

The observed attenuation is therefore unlikely to signify that organizational factors are irrelevant. Between-ward variation in staffing was in fact substantial, with the patient-to-nurse ratio ranging from 6.86 to 14.07 across wards, so the null-adjusted associations cannot be attributed to a homogeneous exposure. Rather, it indicates that aggregated, quasi-static ward-level indicators, assessed over a two-month period, lack sufficient granularity to capture shift-level processes. Workload variability, skill-mix fluctuation and missed nursing care are plausible mechanisms, but none were measured in this pilot; they are advanced here as hypotheses to be tested in the longitudinal phase rather than as established explanations for the present findings. Transferred patients, who had a median stay of 13 versus 7 days and more often came from the emergency department, were also most likely to have been assigned an exposure to a ward other than the one where the event occurred. The lack of adjusted associations may reflect few wards, the aggregation over time, exposure misclassification from using discharge wards, or no real effect. Since transferred hospitalizations differ systematically and ward exposures are continuous, this misclassification is likely not non-differential, and bias direction is unpredictable. This discrepancy aligns directly with the WHO framework, which delineates a strategic domain comprising system-level staffing norms, governance, and financing, and an operational domain involving shift allocation, real-time workload assessment, skill-mix management, and professional judgment. In the complex real-world care environment, these two domains are mutually reinforcing and should be integrated through continuous feedback mechanisms. The longitudinal phase will therefore capture staffing per shift together with a ward-segment table recording each patient’s location with entry and exit times, allowing exposure to be defined as time-varying and linked to the shift preceding each event.

Two further findings are compatible with the case-mix interpretation. First, the counterintuitive bivariate association between a higher number of nursing assistants per shift and outcome occurrence is compatible with confounding by case-mix or patient dependency, although the present data cannot confirm this explanation: higher-acuity wards such as the geriatric and internal medicine wards concentrate both support staff and patients at greater risk. This should not be confused with the documented finding that substituting registered nurses with lower-qualified support staff is associated with higher rates of nursing-sensitive outcomes and mortality [32], a distinction central to the WHO position that support roles should complement, but never substitute for, registered nursing judgment [27]. Second, the medical department showed a crude documented event rate more than twice that of the surgical department (9.71% vs. 4.60%, *p* < 0.001), a difference largely explained by the older and frailer patients admitted to medical wards, frequently from emergency departments. After adjustment for patient-level variables, the direction reversed (OR = 5.67, 95% CI 0.97–33.10; HR = 3.81), but the estimate is imprecise and was not statistically supported once the small number of clusters was accounted for. It should therefore be regarded as hypothesis-generating; if confirmed in an adequately powered sample, it might indicate a residual structural vulnerability in surgical settings related to perioperative immobilization, postoperative pain management, or care transitions to be tested in the longitudinal phase.

Beyond exploratory risk modeling, the principal contribution of this study is to demonstrate that electronic documentation can operationalize the link between staffing and quality that policy now demands. Sharing and using standardized national data can strengthen workforce monitoring through a positive feedback loop, but it requires investment in interoperability and sufficient human resources to ensure accurate, repeatable measurement. Our architecture is a small part of that vision: it provides a foundation on which nursing-sensitive outcomes can be used not merely as retrospective quality indicators but as near-real-time signals informing operational staffing decisions and, when aggregated, to feed strategic governance. Realizing this potential depends on resolving the temporal-resolution mismatch identified here and on capturing real-time acuity, shift-level workload and missed care, which the WHO identifies as the proximal determinants of safe care. Future research should also compare the discriminative performance of the BRASS, a general screening tool for discharge complexity, with outcome-specific scales such as the Braden, Conley or Morse, to establish whether a composite endpoint is better served by a single global explanatory variable or by event-specific instruments.

### Strengths and Limitations

This pilot investigation presents several strengths. To the best of the authors’ knowledge, it represents one of the few real-world demonstrations of an integrated patient- and ward-level digital architecture dedicated to monitoring nursing-sensitive outcomes within an acute care setting in Europe. The employment of routinely collected data resulted in high data completeness without additional documentation tasks at the point of care. Furthermore, the endpoint was operationalized through both association and risk models. These results reflect apparent performance in the development sample and do not indicate readiness for clinical implementation, which would require external validation.

Conversely, several limitations warrant consideration. First, outcome ascertainment relied on documentation-based proxies, specifically the opening of dedicated electronic records, which are susceptible to under-reporting. This under-reporting is likely to be differential rather than random across wards. Consequently, between-ward variation in cumulative event rate confounds true risk, case mix and documentation propensity, and ward-level comparisons are therefore descriptive rather than benchmarks. The organizational variables are constant within wards, so that for these exposures—and for these alone—the effective number of independent units is ten rather than 1441; the corresponding associations are therefore exploratory and their imprecision reflects the number of clusters as well as the effect size. Patient-level variables are estimated from 1441 hospitalizations and are not subject to this constraint, and their associations remain significant under the same small-cluster correction. Repeated admissions for the same individual could not be identified and represent a further unquantified source of statistical dependence. Under-reporting is plausibly most pronounced for physical restraint, which is subject to recognized reporting sensitivities and which, with 14 events, could not support multivariable analysis. Furthermore, pressure injuries present upon admission could not be distinguished from those acquired during hospitalization; since this component accounts for 67 of the 114 events, the composite is dominated by the component with the weakest attributability to ward-level care. A “present-on-admission” field has since been added to the electronic records. Temporal precedence of the BRASS assessment over the event could not be verified: the extraction did not retain the completion time, and 54 of the 114 documented events occurred within the first calendar day, that is, within the window in which the assessment is expected to be performed. Second, patients who declined or could not provide informed consent at admission were excluded, and this exclusion is unlikely to be random: patients with cognitive impairment, delirium or severe clinical instability are both least able to provide valid consent and at highest risk of falls, pressure injuries and, in particular, physical restraint, so the direction of this bias is towards under-estimation of event rate. In addition, the ten included wards were purposively selected based on technical considerations and do not represent a random sample of the twenty units initially planned for inclusion. Admissions involving short-stay, day-surgery, and brief ED observation were excluded in accordance with the study protocol. Because these admissions are characterized by low risk and short duration of exposure, their exclusion may lead to a higher observed cumulative event rate compared to the broader hospital population. Therefore, the reported estimate of 7.35% should not be interpreted as representative of the hospital-wide rate. Third, organizational variables were aggregated using period means, and linkage was performed by discharge ward, potentially introducing exposure misclassification among transferred patients and creating discordance between static structural exposures and dynamic clinical events. Such misclassification affected 15.5% of admissions involving at least one ward transfer. A sensitivity analysis excluding these hospitalizations left the patient-level associations unchanged. Fourth, statistical power was constrained by the limited number of events. This was most evident in the department comparison, whose very wide confidence interval reflects data sparsity and renders that estimate unstable; this association should therefore be interpreted with caution. Adjusted estimates for the department term are consequently reported as exploratory, and the larger event count anticipated in the longitudinal phase will be needed to confirm or refute them. Fifth, the single-center, two-month observational window, confined to the winter season, might limit external validity and be influenced by seasonal variations. Sixth, the exclusion of 111 hospitalizations without a BRASS assessment could have introduced selection bias. These hospitalizations differed by age and sex but not by outcome frequency, admission source or length of stay. The between-ward range in completeness points to documentation practice rather than patient characteristics as the driver of missingness. Multiple imputation across the full sample reproduced the complete-case estimates. The similarity between complete-case and imputed estimates suggests that the principal findings were relatively robust to the handling of missing BRASS data, under the assumptions of the imputation model; it does not establish that selection bias is absent. Seventh, discharge acts as a competing event, so the reported hazard ratios describe rates of occurrence among patients still hospitalized and should not be read as absolute risks. Lastly, the lack of shift-level workload and missed-care metrics precluded the examination of certain operational mechanisms within this pilot framework. The patient-level analysis necessarily omits information regarding event recurrence and cumulative burden, as only the initial event is incorporated into the time-to-event model. Recurrent-event models are therefore planned for the longitudinal phase, wherein the analysis of repeated episodes will be of particular importance. Although no formal sample size calculation was performed, the resulting sample was considered adequate for the pilot study’s predefined objectives on two grounds. First, in terms of precision, the inclusion of 1552 hospitalizations allowed the cumulative event rate to be estimated at 7.35%, with a 95% confidence interval of 6.15–8.75% and a corresponding half-width of 1.30 percentage points. Second, in terms of model estimability, the 114 observed events corresponded to approximately 14.2 events per parameter in models with eight parameters, exceeding the conventional threshold of 10 events per parameter. Nevertheless, the limited study period and number of participating wards precluded reliable component-specific and ward-level analyses. A direct methodological lesson from this pilot is the addition of a structured present-on-admission field to the electronic record, which will allow hospital-acquired pressure injuries to be identified and analyzed separately in the longitudinal phase. As a planning estimate for the longitudinal phase, and not as an expected epidemiological rate, extrapolation of the observed accrual to a 12-month period across the 20 planned wards would yield approximately 18,600 hospitalizations and 1350–1400 events. This projection assumes that accrual in the ten purposively selected pilot wards is representative of the twenty wards planned, which cannot be verified from the present data.

## 5. Conclusions

This pilot study demonstrated that integrating routinely collected electronic clinical records with ward-level organizational data is a feasible strategy for the prospective surveillance of nursing-sensitive outcomes during hospitalization, ensuring high completeness of structured information without imposing additional documentation tasks for ward nurses, within an integrated extraction architecture supported by the hospital information systems department. In this preliminary analysis of 1552 hospitalizations across ten heterogeneous wards, the BRASS and source of admission emerged as the factors most consistently associated with outcome occurrence, whereas ward-level organizational effects could not be reliably estimated with aggregated exposures across ten wards. Collectively, these findings support the operational and initial analytical feasibility of the data architecture developed for this project and delineate priorities for the forthcoming longitudinal phase, which will provide the statistical power and temporal granularity necessary to disentangle structural determinants from case-mix effects on nursing-sensitive outcomes in acute care settings.

## Figures and Tables

**Table 1:** Prespecified feasibility criteria, thresholds and observed results.

Domain	#	Criterion	Prespecified Threshold	Observed Results	Achieved
Operational	i	Automated deterministic linkage of patient- and ward-level data through the unique admission identifier, without manual record matching or supplementary data entry by clinical staff	No ad hoc form completed by ward nurses; no manual matching	No ad hoc form was introduced; linkage was deterministic and automated. Extraction was executed on request by the hospital information systems department, which also validated the output	Yes
Operational	ii	Completeness of essential structured fields (sociodemographic and clinical variables)	≥95%	100% (age, sex, admission source, ward, length of stay, outcome status)	Yes
Operational	iii	Overall BRASS completeness	≥90%, specified as a sample-wide threshold	93.0% overall (ward range 79.3–100%); the threshold would not have been met in 2 of 10 wards had it been applied at ward level	Yes (overall)
Analytical	iv	Events available to support multivariable association models	≥10 events per variable	114 events; 14.2 events per variable for the eight-parameter model	Yes
Analytical	v	Estimability of both the logistic and the time-to-event models, with formal verification of their assumptions	Both models estimable and assumptions formally testable	Both models were estimated; assumptions were formally tested, with a departure from proportional hazards detected for the BRASS	Yes

**Table 2:** Organizational and demographic characteristics of the included wards.

Ward	Beds, *n*	Occupancy Rate	Patient-to-Nurse Ratio	Nurses’ Mean Age	Experience	Education
Cardiology	18	0.96	7.98	42.15	0.48	0.60
Hematology	20	0.95	6.86	45.61	0.67	0.50
Geriatrics	31	1	11.77	40.20	0.14	0.86
Physical Rehabilitation	21	1	7.30	42.32	0.63	0.68
Internal Medicine	30	1	14.07	40.63	0.50	0.69
Oncology	24	0.98	9.17	53.10	0.75	0.17
Pulmonology	29	0.96	9.80	47.13	0.69	0.50
General Surgery	24	0.88	8.17	42.53	0.67	0.80
Orthopedic Surgery	27	0.86	8.20	44.73	0.75	0.69
Urology	28	0.80	8.36	42.06	0.41	0.88

Patient-to-nurse ratio is expressed as the mean value over the observation period; experience is defined as the proportion of nurses with ≥5 years of ward-specific experience; education is intended as the proportion of university-trained nurses.

**Table 3:** Occurrence of nursing-sensitive outcomes across hospital departments.

Department	Total Hospitalizations, *n*	Outcomes, *n*	Cumulative Event Rate Incidence, %	Event Rate Per 1000 Patient-Days at Risk	Event Rate Per 1000 Total Patient-Days
Medical	834	81	9.71	6.01	5.47
Surgical	718	33	4.60	6.25	5.86
Overall	1552	114	7.35	6.08	5.58

Event rate density (time at risk): IRR = 1.04, 95% CI 0.69–1.56, *p* = 0.850. Event rate density (total stay): IRR = 1.07, 95% CI 0.71–1.60, *p* = 0.744.

**Table 4:** Sample characteristics according to the occurrence of nursing-sensitive outcomes.

Variable	No Outcome (*n* = 1438)	Outcome (*n* = 114)	*p*-Value
Age, mean ± SD	70.7 ± 14.4	80.3 ± 12.2	<0.001
Female sex, *n* (%)	525 (36.5)	63 (55.3)	<0.001
Admission source, *n* (%)			<0.001
Emergency Department	741 (51.5)	100 (87.7)	
Waiting List	569 (39.6)	7 (6.1)	
Direct Access	128 (8.9)	7 (6.1)	
Area, *n* (%)			<0.001
Medical	753 (52.4)	81 (71.1)	
Surgical	685 (47.6)	33 (28.9)	
Length of stay, median (IQR), days	7 (3–15)	18 (11–33)	<0.001
BRASS, mean ± SD	7.21 ± 6.75	16.18 ± 8.37	<0.001
Mean bed occupancy rate, mean ± SD	0.91 ± 0.076	0.95 ± 0.061	0.048
Patient-to-nurse ratio, mean ± SD	9.23 ± 1.96	10.33 ± 2.55	0.004

**Table 5:** Multivariable logistic regression for the occurrence of nursing-sensitive outcomes.

Variable	Adjusted OR	95% CI	*p*-Value	Wild Cluster Bootstrap *p*
Surgical department vs. medical department (ref.)	5.67	0.97–33.10	0.054	0.135
Mean bed occupancy rate (per +0.10)	1.93	0.55–6.71	0.303	0.482
Patient-to-nurse ratio (per +1 patient)	1.00	0.89–1.12	0.938	0.953
Age (per +1 year)	1.01	0.99–1.04	0.241	-
Female sex vs. male sex	1.41	0.95–2.07	0.085	-
Admission as direct access vs. waiting list (ref.)	4.15	1.66–10.39	0.002	-
Emergency department admission vs. waiting list (ref.)	4.41	3.02–6.45	<0.001	-
BRASS (per +1 point)	1.12	1.08–1.15	<0.001	-

Complete-case sample = 1441; events = 109. Overall model significance: *p* < 0.001. Odds ratios and confidence intervals are conventional cluster-robust estimates, with standard errors clustered at the ward level (G = 10). Because conventional cluster-robust inference is unreliable with few clusters, ward-level terms were additionally tested using a score-based wild cluster bootstrap with the null imposed, enumerating all 2^10^ = 1024 Rademacher sign combinations; the resulting *p*-values are reported in the final column and are regarded as the primary inference for these terms. Bootstrap confidence intervals are not reported: with ten clusters the bootstrap distribution admits only 1024 distinct sign combinations, so a percentile interval would be too coarse to be informative and could be mistaken for an interval comparable in precision to the conventional one. The attainable two-sided *p*-values are multiples of 1/1024, the smallest being 0.00098. No ward-level association is supported under this correction, whereas the patient-level associations remain significant (BRASS *p* = 0.002; Emergency Department admission *p* = 0.002). The correction applies to ward-level exposures only; patient-level terms are marked “-”.

**Table 6:** Multivariable Cox regression for time to first nursing-sensitive outcome.

Variable	Adjusted HR	95% CI	*p*-Value	Wild Cluster Bootstrap *p*
Surgical department vs. medical department (ref.)	3.81	0.83–17.56	0.087	0.225
Mean bed occupancy rate (per +0.10)	1.36	0.43–4.34	0.602	0.646
Patient-to-nurse ratio (per +1 patient)	1.02	0.92–1.14	0.694	0.791
Age (per +1 year)	1.01	0.99–1.03	0.237	-
Female sex vs. male sex	1.24	0.92–1.68	0.158	-
Admission as direct access vs. waiting list (ref.)	2.59	1.32–5.09	0.006	-
Emergency department admission vs. waiting list (ref.)	3.46	2.48–4.83	<0.001	-
BRASS (per +1 point)	1.10	1.07–1.13	<0.001	-

Complete-case sample = 1441; events = 109. Overall model significance: *p* < 0.001. Hazard ratios and confidence intervals are conventional cluster-robust estimates, with standard errors clustered at ward level (G = 10). Ward-level terms were additionally tested using a score-based wild cluster bootstrap with the null imposed, enumerating all 2^10^ = 1024 Rademacher sign combinations; these *p*-values are reported in the final column and are regarded as the primary inference for these terms. Bootstrap confidence intervals are not reported, for the reason given in the footnote to Table 5. No ward-level association is supported under this correction. The correction applies to ward-level exposures only; patient-level terms are marked “-”.

**Table 7:** Sensitivity analyses of the multivariable logistic model.

Analysis	N/Events	BRASS OR (95% CI)	ED vs. Waiting List	Surgical vs. Medical	AUC
Main model	1441/109	1.12 (1.08–1.15)	4.41 (3.02–6.45)	5.67 (0.97–33.10)	0.828
Excluding events consisting of pressure injuries on admission day	1441/77	1.05 (1.02–1.08)	4.92 (2.72–8.92)	8.61 (1.19–62.40)	0.779
Landmark analysis at 48 h (2 days)	1247/56	1.07 (1.04–1.10)	6.19 (3.77–10.16)	8.93 (1.34–59.25)	0.814
Excluding hospitalization with ward transfers	1229/89	1.11 (1.08–1.15)	3.59 (2.27–5.68)	11.00 (1.03–117.7)	0.830
Composite excluding physical restraint	1441/99	1.12 (1.09–1.15)	3.52 (2.28–5.43)	5.96 (0.68–51.93)	0.826

**Table 8:** Results of the Component-specific Analyses.

Component	Events	Events Per Variable	BRASS OR (95% CI)	Emergency Department vs. Waiting List OR (95% CI)	AUC
Pressure injuries Full model	66	8.2	1.16 (1.12–1.21)	2.81 (1.70–4.66)	0.874
Falls Reduced model	35	8.8	1.05 (1.00–1.10)	5.87 (3.19–10.81)	0.740
Physical restraint Non-estimable model	14	1.8	not estimable	not estimable	-

The full model contains eight parameters; the reduced model contains age, admission source and BRASS.

## Data Availability

The data that support the findings of this study are available from the corresponding author upon reasonable request due to privacy or ethical reasons.

## Supplementary Materials

The following supporting information can be downloaded at: https://www.mdpi.com/article/10.3390/nursrep16090338/s1, Table S1: Comparison of included and excluded hospitalizations with BRASS missing vs recorded.

## Public Involvement Statement

No public involvement in any aspect of this research.

## Guidelines and Standards Statement

This manuscript was drafted against the Strengthening the Reporting of Observational Studies in Epidemiology (STROBE) statement for cohort studies.

## Abbreviations

The following abbreviations are used in this manuscript:

AUC	Area under the curve
BRASS	Blaylock Risk Assessment Screening Score
CI	Confidence interval
ED	Emergency Department
HR	Hazard ratio
ICT	Information and Communication Technology
NDNQI	National Database of Nursing Quality Indicators
OR	Odds ratio
SD	Standard deviation
STROBE	Strengthening the Reporting of Observational Studies in Epidemiology
WHO	World Health Organization
