# Supplementary material for: Feasibility of Using Digital Clinical Records to Identify Factors Associated with Nursing-Sensitive Outcomes During Hospitalization: A Pilot Study
Source: Nurs Rep. 2026 Sep 17;16(9):338. doi: 10.3390/nursrep16090338 (PMC13610447; doi:10.3390/nursrep16090338)
Supplement: Supplementary file 1 [file nursrep-16-00338-s001.zip › nursrep-4501594-supplementary.pdf]

Supplementary Materials

Table S1. Comparison of included and excluded hospitalizations with BRASS missing vs recorded

Characteristic	BRASS missing (n= 111)	BRASS recorded (n= 1441)	p-value
Outcome documented	4.50%	7.56%	0.342
Age, mean (years)	67.9	71.7	0.008
Female	28.8%	38.6%	0.043
Admitted from ED	47.7%	54.7%	0.167
Length of stay, median (days)	7	7	0.866
